# Isolation to stabilization: A Housing First approach to address homelessness in Kingston, Ontario

**DOI:** 10.17269/s41997-024-00936-z

**Published:** 2024-10-15

**Authors:** Yvonne Tan, Zack Revell, Victoria Wilson, T. Hugh Guan, Julie Lambert, Sahar Saeed

**Affiliations:** 1 Department of Medicine, Queen’s University, Kingston, ON Canada; 2 Addiction and Mental Health Services - Kingston, Frontenac and Lennox & Addington (AMHS-KFLA), Kingston, ON Canada; 3 City of Kingston, Kingston, ON Canada; 4 Kingston, Frontenac and Lennox & Addington Public Health (KFL&A), Kingston, ON Canada; 5 Department of Public Health Sciences, Queen’s University, Kingston, ON Canada

**Keywords:** Homelessness, Housing First, Emergency shelter, Substance-related disorders, Mental health, Public health, Sans-abrisme, Logement d’abord, abri d’urgence, troubles liés à une substance, santé mentale, santé publique

## Abstract

**Setting:**

Homelessness is a significant and growing public health concern across Canada. In Kingston, Ontario, the number of people experiencing chronic homelessness has more than doubled from 136 people in 2020 to 296 in 2023.

**Intervention:**

An emergency shelter-in-place hotel program was established in April 2020 to provide non-congregate shelter to people experiencing homelessness and vulnerable to SARS-CoV-2 infections. Beyond preventing COVID transmission, the unintentional consequence was that a population that experienced chronic homelessness reduced drug consumption and became stable. In 2022, with increased funding from the Ministry of Health and the City of Kingston, a new Housing First program was implemented to transition individuals from homelessness to long-term stable housing.

**Outcomes:**

Between November 2022 and June 2023, a total of 34 clients initiated the program. Of these clients, 10 completed the program and were successfully housed, 10 remained active participants, and 14 were discharged before completion. Strengths and challenges were identified. Diverse services provided to meet the population’s needs and strong collaborations with various community partners were facilitating factors. Inadequate external resources, a lack of evening and prosocial activities, and outside peers (not part of the program) who influenced recovery plans were identified as challenges.

**Implications:**

This program illustrates that simultaneously integrating housing, community building, mental health, and addiction services is possible and provides an innovative way to stabilize this vulnerable population of people experiencing homelessness. Results from this program and the knowledge generated through implementation are being used to further scale up the program.

## Résumé

**Lieu:**

Le sans-abrisme est un problème de santé publique important et en croissance au Canada. À Kingston, en Ontario, le nombre de personnes aux prises avec le sans-abrisme chronique a plus que doublé entre 2020 et 2023, passant de 136 à 296 personnes.

**Intervention:**

Un programme d’abri d’urgence à l’hôtel mis en place en avril 2020 fournit un abri non collectif aux personnes aux prises avec le sans-abrisme vulnérables aux infections par le SRAS-CoV-2. En plus de prévenir la transmission de la COVID, ce programme a eu un effet inattendu : la population aux prises avec le sans-abrisme chronique a réduit sa consommation de drogue et s’est stabilisée. En 2022, grâce à un financement accru du ministère de la Santé et de la Ville de Kingston, un nouveau programme (Logement d’abord) a été mis en œuvre pour faciliter la transition des personnes sans abri vers un logement stable à long terme.

**Résultats:**

Entre novembre 2022 et juin 2023, 34 personnes se sont inscrites à Logement d’abord. Dix d’entre elles ont terminé le programme et ont été logées avec succès, 10 y participent encore activement, et 14 en sont sorties avant la fin. Nous avons cerné les forces et les limites du programme. Les facteurs favorables ont été la gamme de services fournis en réponse aux besoins de la population et les liens de collaboration solides avec divers partenaires associatifs. Les limites du programme ont été le manque de ressources externes, l’absence d’activités prosociales et en soirée et l’influence des pairs de l’extérieur (non inscrits au programme) sur les plans de rétablissement des usagères et des usagers.

**Conséquences:**

Ce programme montre qu’il est possible d’intégrer simultanément le logement, la solidarité sociale, la santé mentale et les services en toxicomanie, et que c’est un moyen novateur de stabiliser cette population vulnérable aux prises avec le sans-abrisme. Les résultats du programme et les connaissances apportées par sa réalisation serviront à le reproduire à plus grande échelle.

## Introduction

Homelessness is a multifaceted issue affecting millions worldwide (Fowler et al., 2019). In Canada, an estimated 235,000 people experience homelessness yearly, with an estimated 35,000 experiencing homelessness on any night (Baral et al., 2021). Housing is a fundamental social determinant of health, given that homelessness exacerbates various health risks, including mental illness, substance use disorder, infectious diseases, chronic health conditions, and mortality (Hwang, 2001).

It is well documented that adverse life events such as physical abuse, foster care, and psychiatric illness have been associated with an increased risk of homelessness (Nilsson et al., 2019). In particular, alcohol-use disorders and drug-use disorders have been found to increase prospective risk for first-time homelessness (Thompson et al., 2013).

## What is Housing First?

Recognizing the intricate connection between homelessness and substance use, Housing First has emerged as a promising solution. Housing First is rooted in the understanding that, without stable housing, it is difficult for individuals to address underlying issues—including substance use—that may have impacted their housing status. By combining stable housing with intensive services, Housing First aims to facilitate long-term stability, improve health outcomes, and enable successful reintegration into society (Kneebone & Jadidzadeh, 2022).

New York City’s 1992 Pathways to Housing was the original Housing First model; it has produced several studies demonstrating its success. A randomized controlled trial comparing Pathways to Housing with a control group found that clients in the Housing First trial group reported significantly higher percentages of time spent housed at 6, 12, 18, and 24 months (Tsemberis et al., 2004). A subsequent study revealed that, in the same 225 participants, those randomly assigned to the experimental condition spent significantly less time in psychiatric hospitals and incurred fewer costs than controls (Gulcur et al., 2003). In Canada, the At Home/Chez Soi project was a 4-year randomized controlled trial that evaluated a Housing First intervention in Vancouver, Winnipeg, Toronto, Montreal, and Moncton. Across all cities, Housing First participants obtained housing and retained their housing at a much higher rate than the treatment-as-usual group (Goering et al., 2014). These studies have provided evidence to support Housing First, leading numerous cities across North America and Scandinavia to adopt such programs (Baxter et al., 2019; Mares & Rosenheck, 2011; McGraw et al., 2010).

Despite these optimistic outcomes, it is important to note that the vast majority of studies on Housing First have occurred in the United States (Ecker et al., 2014). Studies that have been conducted in Canada have focused primarily on major urban centres. A recent review highlighted there has been a notable lack of research on mid-sized, northern, suburban, and small communities (Smith & Kopec, 2023) despite the potential unique experiences of homelessness in such populations (Whitzman, 2006).

## How have hotels been used in housing programs?

Hotels and motels have been used for decades to temporarily house people experiencing homelessness (Brickner, 1972; Grant, 1991; Plutchik et al., 1975). However, the COVID-19 pandemic led to a novel use of hotel programs across North America. In New York City, over 9000 congregate shelter residents were moved into hotel rooms; likewise, Los Angeles County moved 4300 individuals experiencing homelessness into hotels (Padgett et al., 2022). San Francisco’s COVID-19 shelter-in-place hotel program provided temporary housing to 3877 in private hotels, motels, and trailers (Abbs et al., 2023). Such programs not only decreased the spread of COVID-19 (Colburn et al., 2022; Huggett et al., 2021; Montgomery et al., 2021), but also led to decreased use of medical services (Fleming et al., 2022; Fuchs et al., 2021), increased stability (Colburn et al., 2022; Padgett et al., 2022; Robinson et al., 2022), and improved health and well-being (Colburn et al., 2022; Huggett et al., 2021; Padgett et al., 2022).

Nevertheless, as with other Housing First programs, studies on such hotel programs have focused on large urban centres in the United States.

## Implementation of Kingston’s Stabilization Program

### Setting

Kingston, Ontario is a medium-sized city with a population in 2021 of 172,546. The majority of Kingston residents (84.2%) identify as white or European, while 10.1% identify as visible minorities, and 5.7% as Indigenous (Statistics Canada, 2017). Uniquely, five of Ontario’s eight correctional institutions are located in the Kingston area, creating a transient population (Government of Canada, 2013). Based on the city of Kingston’s homeless registry, approximately 300 individuals experienced chronic homelessness, defined as those who were unsheltered and those staying in emergency or domestic violence shelters (*Homelessness Registry By-Name List*, 2023). Overall shelter capacity in 2021 was down to 67 beds, compared to the pre-COVID capacity of 94 beds (United Way – Kingston, Frontenac Lennox and Addington, 2021). The average market rent in Kingston is higher than that for cities of similar size and rental rates are disproportionate to the housing allowances for social assistance (United Way – Kingston, Frontenac Lennox and Addington, 2021).

### Program description

In April 2020, the Addiction and Mental Health Services—Kingston, Frontenac and Lennox & Addington (AMHS-KFLA) implemented an emergency hotel program to provide non-congregate shelter to people experiencing homelessness and vulnerable to SARS-CoV-2 infections. With funding from the Ontario Ministry of Health and the City of Kingston, a section of a local motel was repurposed to provide 10 beds of isolated shelter, with clients staying in the program for approximately 14 days.

Beyond preventing COVID-19 transmission, the unintentional benefit was that the individuals who had been chronically unhoused for years became stable. The goal of the program in Kingston therefore evolved from isolation to stabilization. However, staff identified challenges with completing referrals, building therapeutic rapport, and developing support plans within the short 14-day window. Additional funding was therefore granted by both the Ministry of Health in August 2022 and the City of Kingston in November 2022 to allow the new Stabilization Program to service up to 15 beds for stays up to approximately 9 months, with access to addictions and mental health supports and staff on-site. Each client has their own private motel room and access to shared indoor and outdoor facilities. Daily services are provided by the program, including arts and crafts, life skills, understanding addictions, recreation, and leisure.

The Stabilization Program currently occupies 18 rooms in the motel, with 15 rooms for clients, one office room for staff, and two community rooms. Staff include four full-time case managers, a registered nurse, a team lead, and a program manager.

Currently, clients who are referred to the Stabilization Program are those who are experiencing homelessness, using substances however expressing motivation to work towards recovery, and capable of adhering to programming. Referrals are accepted internally from AMHS-KFLA Street Outreach and externally from the Kingston Community Health Centres (KCHC) Street Health Centre and the Hotel Dieu Hospital Substance Treatment and Recovery Team (START) Program.

### Characteristics of clients

Before evaluating this program, ethics approval was obtained from the Health Sciences Research Ethics Board (HSREB) at Queen’s University. Table 1 describes the 34 clients enrolled in the Stabilization Program from August 15, 2022, to June 30, 2023. The median age was 42 (IQR 35–48); 71% self-identified as male, 82% as non-Indigenous, and 6% as being of a visible minority; 21% had less than a high school education; and 100% were English-speaking. The main sources of income were the Ontario Disability Support Program (ODSP) (76%) and Ontario Works (15%). All clients (100%) had previously accessed other housing or addictions services, and the median experience of homelessness was 21 months (IQR 11–33).

**Table 1:** Baseline demographic composition of Stabilization Program clients at intake

	*N* (%) or median (IQR)
Age (years)	42 (35, 48)
Gender	
Male	25 (71%)
Female	9 (29%)
Cumulative time spent experiencing homelessness (years)	17 (11, 33)
Number of times unhoused	2 (1–4)
Indigenous status	
Indigenous	6 (18%)
Non-Indigenous	28 (82%)
Identify as visible minority	
Yes	2 (6%)
No	32 (94%)
Marital status	
Single	32 (94%)
Divorced	2 (6%)
Highest level of education	
Less than high school	7 (21%)
High school	7 (21%)
Some college or university	8 (24%)
Unknown	11 (33%)
Income source	
ODSP	26 (76%)
Ontario Works	5 (15%)
Employed	1 (3%)
Employment insurance	1 (3%)
None	1 (3%)
Mental health diagnoses (clients may have more than one)	
Borderline personality disorder	4 (12%)
Anxiety/depression	8 (24%)
Schizophrenia	8 (24%)
Psychosis	8 (24%)
PTSD	3 (​​9%)
Other	14 (41%)
Previously received mental health treatment	20 (59%)
Self-identify as requiring mental health support	19 (56%)
Substance of choice	
Marijuana	9 (26%)
Crystal methamphetamine	31 (91%)
Cocaine	4 (12%)
Benzodiazepines	1 (3%)
Fentanyl	12 (35%)
Alcohol	5 (15%)
Duration of substance use (years)	
Marijuana	30 (18, 30)
Crystal methamphetamine	8.5 (4, 10)
Cocaine	1 (4, 13)
Benzodiazepines	Not enough data
Fentanyl	5 (2, 6)
Alcohol	35 (33, 44)
Use of opiate replacement therapies	17 (50%)

Four in five clients self-reported mental illnesses, including a range of anxiety disorders, mood disorders, personality disorders, and psychotic disorders. Yet only 59% reported a history of receiving mental health treatment. Prior to entering the program, 56% identified a need for mental health support and 43% reported having a learning disability. As an eligibility criterion, all clients reported active substance use; the majority of clients reported using crystal methamphetamine (91% for a median of 8.5 years), fentanyl (35% for a median of 5.0 years), and marijuana (26% for a median of 30.0 years). Half of the clients reported being treated with opiate replacement therapies.

Many had accessed various healthcare services before initiating the program, including the emergency department (82%) and inpatient care (24%). Additionally, 94% of clients had prior interactions with the criminal justice system.

## Evaluation of the Stabilization Program

### Outcomes

As of July 2023, of the 34 clients who initiated the program, 10 completed the program and were successfully housed, while 11 were discharged (either voluntarily or by staff due to an inability to comply with program policies and procedures), 1 completed the program without housing, 1 declined intake into the program, 1 was incarcerated, and 10 were still enrolled in the program at the time of evaluation. Those who completed the program stayed the full 9 months, while those who were discharged left between 1 and 3 weeks after program initiation.

*All my needs have been met and then some. The help and attention that has been shown and given to me has been way more than I expected. (Client)*

### Enabling factors

#### Internal facilitators

Program administrators identified the comprehensive services provided by the Stabilization Program as an enabling factor. Many of the pre-existing housing programs in Kingston were focused solely on housing and lacked services to also address addiction and mental health issues. In comparison, Stabilization Program staff were on site 7 days a week, which allowed for daily programming including developing skills to manage urges to use substances; increasing prosocial activities; and collaborating with local housing services to support with finding independent housing.

*This kind of program is very new in the province - it is a combination of a mental health and addictions service and a housing service. [...] It’s somewhere between a healthcare solution and a housing solution. (City of Kingston representative)*

Offering the program for 9 months was another important strength. Housing programs currently in place in Kingston consist largely of emergency shelters and drop-in services. The Stabilization Program instead presented a holistic solution that bridged the gap between emergency shelters and long-term residential treatment, allowing for relationships of trust to be built with clients as well as moving clients along the housing continuum.

*It’s not just keeping somebody safe and healthy, but it’s also moving somebody along in their path to recovery. (City of Kingston representative)*

#### External facilitators

An important external factor that enabled the implementation of the program was community engagement. Collaboration with interdisciplinary community partners was identified as critical to program success. Many of the clients accessing the Stabilization Program were previously connected with other community agencies—for example, several are on the city’s homelessness registry (a comprehensive data source of every person in a community experiencing homelessness). Such ongoing service planning was beneficial for ensuring consistency of treatment. Continuous engagement in meetings with community members (including people experiencing homelessness), local housing services, and addictions services enhanced program development. Many social and health services in Ontario are decentralized and municipal in nature, which facilitated strong engagement between the KFL&A Public Health and the City of Kingston—for example, through the Mayor’s Task Force on Housing Committee and the Homelessness Collective Impact Committee.

### Limiting factors

#### Internal barriers

A lack of staffing was noted to be a limiting factor. The location and space of this program was limited to only 15 beds, but staffing capacity could only support 10 clients. Additionally, the Stabilization Program was only staffed until 7:00 pm Monday to Thursday, until 5:00 pm on Friday, and until 1:00 pm on weekends. There were no groups or programs offered in the evening, but it was noted that evening programming may add more structure, routine, and support with building healthy relationships.

#### External barriers

There are limited affordable housing options in Kingston for those who rely solely on social assistance or disability benefits. Housing options that are both affordable and available are often shared accommodations or room rentals; with such accommodations, there is a risk that other residents may negatively influence graduates from the Stabilization Program.

Another external factor was an inability for program staff and participants to limit access to the environment. The building was a motel, which allowed individuals not participating in the Stabilization Program to encounter and potentially influence or exploit program participants. This was identified to be potentially compromising, since external people may put the clients in situations that could hinder their progress.

## Discussion

As the Stabilization Program continues to make a meaningful impact in the lives of people experiencing homelessness, a collection of key insights has emerged.

With regard to internal factors enhancing program implementation, embedded addictions and mental health services were key to promoting recovery and empowering participants. Programming to keep clients engaged and occupied served to provide clients with life skills support, which has been found to be essential for sustained recovery (Ponce et al., 2018; Stergiopoulos et al., 2014). Programming also offered a venue for clients to meet one another; peers serve as role models, accountability mechanisms, and sources of encouragement, enhancing overall engagement and progress (Cummings et al., 2022; Stergiopoulos et al., 2014). Furthermore, longer stays allowed for a deeper connection between participants and staff, an important factor in improving support and care for people experiencing homelessness (Ponce et al., 2018; Stona et al., 2016).

External factors should also be considered. Building positive relationships with various community partners enriched the program’s offerings and enhanced its credibility and impact within the community; partnerships facilitate buy-in and engagement as well as foster a supportive ecosystem extending beyond the program’s immediate scope (Ponce et al., 2018; Worton et al., 2018). However, individuals seeking to exploit program clients must also be considered—the vulnerable nature of individuals experiencing homelessness and substance use disorders makes them susceptible to exploitation by people selling drugs and others looking to take advantage of their circumstances. Distance from such negative influences has been found to enhance recovery and promote stable housing outcomes (Cummings et al., 2022).

A limitation of this study is that it describes a program that benefited from dedicated funding resources. While this financial support facilitated the program’s implementation, the generalizability of the findings to contexts with differing resources may be limited.

Following its initial success, the Stabilization Program will move to a larger building with a range of amenities and widen its scope of accepted referral sources. Community partnerships will remain at the heart of this expansion, in hopes of promoting a wraparound network of support that is tailored to the diverse needs of the individuals served.

## Implications for policy and practice

What are the innovations in this policy or program?

- The Ministry of Health and City of Kingston implemented a hotel program to support people experiencing homelessness and actively using substances in transitioning to long-term stable housing and reducing consumption. The program was considered a success, and funding was renewed.
- The Stabilization Program had several advantages compared to pre-existing services as it embeds addictions and mental health services, provides intermediate-term housing to help clients move through the housing continuum, and collaborates with community partners in a medium-sized city.

What are the burning research questions for this innovation?

- Will graduating from this program reduce healthcare service utilization and encounters with the justice system?
- Is the program cost-effective?
- How effective will this program be as it is scaled up? And what will the ongoing challenges be?
- How can this program be adopted and modified by another community, considering individual and unique community contexts?

## Data Availability

Due to the confidential nature of this study, the data are not publicly available.
